# Intraspecific functional and genetic diversity of *Petriella setifera*

**DOI:** 10.7717/peerj.4420

**Published:** 2018-02-28

**Authors:** Giorgia Pertile, Jacek Panek, Karolina Oszust, Anna Siczek, Magdalena Frąc

**Affiliations:** Institute of Agrophysics, Polish Academy of Sciences, Lublin, Polska

**Keywords:** Genetic fingerprinting, *Petriella setifera*, Metabolic diversity, Soft-rot fungi

## Abstract

The aim of the study was an analysis of the intraspecific genetic and functional diversity of the new isolated fungal strains of *P. setifera*. This is the first report concerning the genetic and metabolic diversity of *Petriella setifera* strains isolated from industrial compost and the first description of a protocol for AFLP fingerprinting analysis optimised for these fungal species. The results showed a significant degree of variability among the isolates, which was demonstrated by the clearly subdivision of all the isolates into two clusters with 51% and 62% similarity, respectively. For the metabolic diversity, the BIOLOG system was used and this analysis revealed clearly different patterns of carbon substrates utilization between the isolates resulting in a clear separation of the five isolates into three clusters with 0%, 42% and 54% of similarity, respectively. These results suggest that genetic diversity does not always match the level of functional diversity, which may be useful in discovering the importance of this fungus to ecosystem functioning. The results indicated that *P. setifera* strains were able to degrade substrates produced in the degradation of hemicellulose (D-Arabinose, L-Arabinose, D-Glucuronic Acid, Xylitol, γ-Amino-Butyric Acid, D-Mannose, D-Xylose and L-Rhamnose), cellulose (α-D-Glucose and D-Cellobiose) and the synthesis of lignin (Quinic Acid) at a high level, showing their importance in ecosystem services as a decomposer of carbon compounds and as organisms, which make a significant contribution to carbon cycling in the ecosystem.The results showed for the first time that the use of molecular biology techniques (such as AFLP and BIOLOG analyses) may allow for the identification of intraspecific diversity of as yet poorly investigated fungal species with favourable consequences for our understanding their ecosystem function.

## Introduction

The species of *Petriella setifera* (Alf. Schmidt) Curzi belongs to the family Microascaceae of the division Ascomycota, Kingdom Fungi, and is commonly found in enriched soil (for example dung, manure, or composts) (Danon, Chen & Hadar, 2010; Lackner & De Hoog, 2011) or in the teleomorph stage on dung or decayed wood as soft-root fungi (Issakainen et al., 1999). *P. setifera* was also isolated from twigs of sessile oak (*Quercus petraea* (Mattuschka) Liebl.) (Kwaśna, Łakomy & Łabędzki, 2005), the roots of *Pinus roxburgii* Sarg., *Nothapodytes nimmoniana* (J. Grah.) D.J. Mabberley (Qadri et al., 2013), and *Salvia miltiorrhiza* Bunge (Lou et al., 2013).

The family Microascaceae consists of 20 genera and 200 species. In this family, a limited number of fungi potentially harmful or infectious to humans may be found (which are from the *Pseudallescheria* and *Scedosporium* genus) (Rainer & De Hoog, 2006). In fact, when Issakainen et al. (1999) analysed the *Petriella* clade (based on LSU rDNA) this analysis included the human pathogenic *Scedosporium prolificans*. However, the Sordariomycetes class at the level of taxonomic relationships and classifications remains more obscure (Tang, Jeewon & Hyde, 2007). The molecular analysis of this class began in 1990 through a study of the LSU and SSU regions of rDNA. The ITS, LSU, and SSU regions are good indicators of phylogeny analysis (Tang, Jeewon & Hyde, 2007).

From the point of view of the ecosystem function, *P. setifera* is very important because of the possible role in nutrient cycling, wood decay and also as an opportunistic antagonist (Danon, Chen & Hadar, 2010); it also belongs to the fimicolous fungi group and produces secondary metabolites that engage in antimicrobial activity against other microorganisms (Umehara et al., 1983). The fungi that belong to the fimicolous group live optionally on dung but they are also able to survive and develop on other substrates (Sarrocco, 2016). These fungi produce secondary metabolites which may be used in nature (as a form of biological control), in agriculture (as natural pesticide), and medical fields (as antibiotics). Furthermore, these metabolites may influence the life or growth of other microorganisms. In particular, *Petriella* fungi can produce an antitumour antibiotic (Umehara et al., 1983) and a Petriellin A (Bills, Gloer & An, 2013), which has been reported to be an antifungal agent against *Sordaria fimicola* and *Ascobolus furfuraceus* (Sarrocco, 2016). However, the *Petriella* genus is composed of more species, including *P. setifera*, which have a different function inside the ecosystem and are involved in biochemical and nutrient cycles. Some of these species are classified as decomposers, as they play an important role in the carbon and nitrogen cycles. For this reason, the exploration and research of the coprophilous and fimicolous fungi have led to their biodiversity analysis.

The work presented includes currently accepted concepts in the relationship between diversity and functioning of the fungi, as well as details concerning the importance of *Petriella setifera* to ecosystem function through an analysis of the genetic diversity and metabolic profile of this fungal species. What is more, due to a the lack of information concerning the intraspecific diversity and functionality of *P. setifera* in the soil and organic waste, the research provided enhances our knowledge concerning the services of this fungus to the ecosystem by exploring their functional and genetic profiles. The study of the genetic and functional attributes of *Petriella*, serves to fill a major knowledge gap concerning this fungi and wood degradation as well as ecosystem carbon cycling in general. The metabolic and genetic characterization of *Petriella setifera*, as a representatives of the Microascaceae family, will enhance our knowledge concerning these fungi as important coprophilous players in soil food webs. In the present work, since there is insufficient information about the functional genetic diversity and metabolic potential of *Petriella* sp., we use these analyses to evaluate the genetic and functional diversities between *Petriella setifera* strains isolated from compost with the final of aim to explaining the role of these fungi in ecosystem functioning and to find the intraspecific differences among these isolates without possessing any genetic information about the analysed species. To determine the genomic variability, we propose an analysis of the AFLP fingerprinting; in turn, we propose the analysis of the fungal ability to use different carbon sources using the BIOLOG FF MicroPlates™ system to determine its metabolic potential. In this paper, we have demonstrated for the first time a combination of genomic and functional diversity assays to determine the role of *P. setifera* in the ecosystem overall. Moreover, the first protocol of AFLP fingerprinting analysis applied to this species was developed within this study.

## Materials and Methods

### *Petriella setifera* isolates

Five strains of *P. setifera* (G11/16; G14/16; G16/16; G17/16; G18/16) were isolated from industrial compost using the serial dilutions method on Bengal Rose LAB-AGAR (BIOCORP, Warszawa, Poland).

The compost consisted of the following organic substances: sewage sludge from wastewater treatment, sawdust, biodegradable garden and park waste, soil, mouldings of medicinal plants obtained by solvent extraction, and lime sludge. The concentrations of the principal components of the compost, i.e., total carbon, nitrogen and phosphorus were respectively 17.9%, 2.3%, and 0.75%, respectively and the pH was 5.3.

### Fungal DNA extraction

The analysed strains were cultured on 90 mm Petri dishes with a Potato Dextrose Agar medium (Oxoid Ltd, Basingstoke, UK) at 30 °C for 14 days. 200 mg of fungal mycelium was taken from each of the five strains and sterilely transferred into 2 ml tubes containing 250 mg of glass beads of 1.45 mm diameter and 500 mg of glass beads of 3.15 mm diameter and they were homogenized with a FastPrep-24 homogenizer (MPBio, Santa Ana, CA, USA) at 4 m/s for 20 s. The DNA was extracted in accordance with the EURx GeneMATRIX Plant and Fungi DNA Purification Kit (EURx, Gdańsk, Poland) protocol. The quantity and purity of the extracted DNA were evaluated with a NanoDrop-2000 Spectrophotometer (Thermo Scientific, Waltham, MA, USA).

### D2 LSU rRNA and ITS1 regions sequencing

The sequencing of the D2 LSU rRNA and ITS1 regions were performed with the use of primers designed by us and also with the use of universal primers (Table 1).

**Table 1:** The list of oligonucleotide primers used in sequencing of the D2 region of LSU rRNA.

Primer name	Primer sequence	Reference
D2LSU2_F	5′-AGA CCG ATA GCG AAC AAG-3′	This study
D2LSU2_R	5′-CTT GGT CCG TGT TTC AAG-3′	This study
ITS1	5′-TCC GTA GGT GAA CCT GCG G-3′	White et al. (1990)
ITS2	5′-GCT GCG TTC TTC ATC GAT GC-3′	White et al. (1990)

The primary amplification of the target D2 LSU rRNA was performed in a final volume of 20 µl in a Veriti Fast thermal cycler (Applied Biosystem, Foster City, CA, USA). Each reaction contained 10 µl of 2X PCR Reaction Master Mix (EURx, Gdańsk, Poland), 1 µl of DNA template, 1 µl of 10 µM D2LSU2_F primer, and 1 µl of 10 µM D2LSU2_R primer. The reactions were set up as follows: 95 °C for 600 s followed by 35 cycles at 95 °C for 15 s, 53 °C for 20 s, and 72 °C for 20 s, and followed by a final step at 72 °C for 300 s. The target ITS1 analysis was performed in a final volume of 20 µl in a Veriti Fast thermal cycler (Applied Biosystem, Foster City, CA, USA). Each reaction contained 10 µl of 2X REDTAq^®^ReadyMix PCR (Sigma-Aldrich, St. Louis, MO, USA), 2 µl of DNA template, 0.2 µl of 20 µM ITS1 primer, and 0.2 µl of 20 µM ITS2 primer. The reactions were set up as follows: 94°C for 180 s followed by 35 cycles at 94 °C for 15 s, 55 °C for 30 s, and 76 °C for 40 s, and followed by a final step at 76 °C for 420 s.

At the end of this reaction, 5 µl of products were purified with exonuclease I–bacterial alkaline phosphatase, by mixing with 2 µl of Exo-BAP Mix (EURx, Gdańsk, Poland). The samples were then incubated at 37 °C for 15 min and afterwards at 80 °C for another 15 min. In the following step, the samples were diluted 1:10 with sterile water. The sequencing reactions were performed in a final volume of 10 µl containing 0.5 µl of BigDye^®^ Terminator v1.1 Reaction Mix (Thermo Fisher Scientific, Waltham, MA, USA), 2 µl of sequencing buffer (400 mM Tris, 10 mM MgCl_2_, pH 9.0), 1 µl of 3.33 µM D2LSU2_F or D2LSU2_R primer, 0.17 µl of 20 µM ITS1 or 0.17 µl of 20 µM ITS2 and 1 µl of diluted PCR product. The reactions were performed using the specified conditions: 96 °C for 60 s followed by 45 cycles at 96 °C for 10 s, 50 °C for 5 s, 60 °C for 120 s for D2 LSU or 60 °C for 180 s for ITS1 sequencing. Subsequently, all samples were purified with Performa^®^ DTR cartridges (Egde BioSystem, Gaithersburg, MD, USA). The purified products were mixed with 10 µl of HiDi formamide (Applied Biosystems, Foster City, CA, USA) and incubated at 95 °C for 180 s followed by 4 °C for 180 s; next, they were loaded into the Applied Biosystems 3130 Genetic Analyser (Applied Biosystems, Foster City, CA, USA) with a 50 cm capillary array filled with NanoPOP-7 Polymer (McLAB, South San Francisco, CA, USA).

Sequences of all strains were deposited in the National Centre for Biotechnology Information (NCBI; http://www.ncbi.nlm.nih.gov) (Woodsmall & Benson, 1993) under the following accession numbers: KX639331, KX639334, KX639335, KX639336, and KX639337, and MG594608.1, MG594609.1, MG594610.1, MG594611.1, and MG594612.1 for LSU rDNA and ITS region, respectively.

### AFLP analysis

The AFLP reactions were performed with the use of *Pst*I and *Mse*I restriction enzymes. The results of the analysis were visualised by capillary electrophoresis with the Applied Biosystems 3130 Genetic Analyser (Applied Biosystems, Foster City, CA, USA). The sequences of the adapters and primers used in this study are shown in Table 2.

**Table 2:** The list of oligonucleotide primers and adapters used in AFLP analysis.

Adaptor name	Adaptor sequence 5′–3′
*Mse*I_AF	GAC GAT GAG TCC TGA G
*Mse*I_AR	TAC TCA GGA CTC AT
*Pst*I_AF	CTC GTA GAC TGC GTA CAT GCA
*Pst*I_AR	TGT ACG CAG TCT AC

Primer name	Primer sequence 5′–3′
6-FAM-*Pst*I + ACA	FAM-GAC TGC GTA CAT GCA GAC A
*Mse*I + CA	GAT GAG TCC TGA GTA ACA

The AFLP reactions were performed in three biological replications for each isolate. The double-stranded *Pst* I and *Mse* I oligonucleotide adapters were formed in a final volume of 2 µl by incubating 0.5 µl of 10 µM *Pst* I_AF, 0.5 µl of 10 µM *Pst* I_AR, 0.5 µl of 100 µM *Mse* I_AF, and 0.5 µl of 100 µM *Mse* I_AR adapters at 95 °C for 5 min followed by 15 min at room temperature. Next, the restriction-ligation (RL) reaction was performed. The genomic DNA (500 ng) was digested with 5 U of the *Pst* I restriction enzyme (EURx, Gdańsk, Poland) and 5 U of the *Mse* I restriction enzyme (New England Biolabs, Ipswich, MA, USA). The RL solution was composed of 1 U of T4 DNA Ligase (EURx, Gdańsk, Poland), 2 µl of double-stranded adapters, 50 mM Tris–HCl, 10 mM MgCl_2_, 10 mM DTT, 1 mM ATP, and 25 µg/ml of BSA in a final volume of 20 µl. The RL reaction was incubated for 1 h at 37 °C. At the end of this reaction, each RL reaction mixture was diluted with the addition of 80 µl of sterile water and 1 µl of this solution was used as a template in the selective amplification reaction. The selective PCR amplification reaction was performed in a final volume of 5 µl, which consisted of 2.5 µl of 2X Taq PCR Reaction Master Mix (EURx, Gdańsk, Poland), 1 µl of diluted RL solution, 0.25 µl of 10 µM 6-FAM-*Pst* I+ACA primer (Genomed, Poland), and 0.25 µL of 10 µM *Mse* I+CA primer (Genomed, Warszawa, Poland). The reaction was performed in a Veriti Fast thermal cycler (Applied Biosystems, Foster City, CA, USA) under the following conditions: 72 °C for 120 s followed by 7 cycles at 94 °C for 15 s, 63 °C with a touchdown of −1 °C per cycle for 30 s, 72 °C for 45 s followed by 33 cycles of 94 °C for 45 s, 56 °C for 30 s, 72 °C for 45 s, and followed by a final step at 72 °C for 60 s. At the end of this step, the purification of exonuclease I–bacterial alkaline phosphatase was performed by the addition of 2 µl of Exo-BAP Mix (EURx, Gdańsk, Poland) to each reaction tube. The samples were incubated at 37 °C for 15 min and then at 80 °C for another 15 min. In the next step, 28 µl of sterile water was added into each PCR-product and 0.5 µl of this solution was combined with 0.25 µl of GS-600 LIZ Standard (Applied Biosystems, Foster City, CA, USA) and 9.25 µl of HiDi formamide (Applied Biosystems, Foster City, CA, USA). This mixture was incubated for 150 s at 95 °C and cooled down using ice for 5 min. The amplicons were separated by capillary electrophoresis with the Applied Biosystems 3130 Genetic Analyser (Applied Biosystems, Foster City, CA, USA) in a 50 cm capillary array filled with NanoPOP-7 Polymer (McLAB, South San Francisco, CA, USA). The fragments were compared to the standard and visualized as an electropherogram with GeneMapper^®^ version 4.0 software (Applied Biosystems, Foster City, CA, USA).

### Fungal isolate phenotype profiles (FIPPs)

The phenotype profiles of *Petriella setifera* isolates, regarding their catabolic potential, were generated based on the organism growth intensity on 95 substrates located on BIOLOG FF plates (Biolog Inc., USA) at low-molecular weight carbon sources.

The inoculation procedure was based on the original FF microplate (BIOLOG™) method according to the manufacturer’s protocol modified by Frąc (2012). To prepare the inoculum, mycelia of each isolate were obtained by cultivation on Potato Dextrose Agar medium (Oxoid Ltd, England) in the absence of light at 30 °C for 10 days. The transmittance of the mycelium homogenized suspension in inoculating fluid (FF-IF, BIOLOG™) was adjusted to 75% using a turbidimeter (BIOLOG™). Then, 100 µl of the mycelium suspension was added to each well and the inoculated microplates were incubated at 26 °C for 10 days. The experiment was carried out in two biological replications. The optical density at 750 nm was determined in triplicates using a microplate reader (BIOLOG™) every day. Functional diversity was determined by the number of different substrates utilized by the individual isolates and expressed as the substrate richness (R) and Average Well Density Development (AWDD) indices. The AWDD index was determined through the optical density of each well corrected by the subtraction of the blank (water) divided by the total number of wells (95-wells).

### Statistical analysis

The sequences, which they were obtained from the Applied Biosystems 3130 Genetic Analyser (Applied Biosystems, Foster City, CA, USA), were analysed with a Sequence Analysis program (Applied Biosystem, Foster City, CA, USA) and with the MEGA version 10.0 software (Kumar, Stecher & Tamura, 2016); an alignment by the MUSCLE algorithm (Edgar, 2004) was made. The evolutionary history was inferred by using the Maximum Likelihood method based on the Tamura-Nei model (Tamura & Nei, 1993). Moreover, in the dendrogram we have included additional sequences of the fungal species, which have already been published, whether or not they belong to the same *Petriella setifera* family. This process has been completed in order to identify with certainty, the five fungal strains isolated from industrial compost and both to compare the *P. setifera* strains with others published fungal genomes.

To illustrate the BIOLOG results, the similarities of the carbon utilization patterns between the strains were presented using heatmaps graphs and the percentage of total carbon source utilization. For the substrate, the richness (R) and AWDD indices were assessed, by two-way ANOVA analysis regarding the effect of the incubation time and the type of strain. Successively, the significant differences were calculated by a post hoc analysis using the Tukey test. As a function of the carbon utilization, we performed a cluster analysis using a dendrogram calculated with the Ward method and Sneath’s dissimilarity criterion which was calculated using the function of the dissimilarity of fungal groups on the basis of their response to standard tests (Sneath & Sokal, 1973).

On the other hand, for the AFLP results, we only considered only the peaks of amplified fragments that are longer than 200 bp and have area an parameter higher than 1,000. We assigned value 1 for the presence of a peak and value 0 for the absence of a peak. The results obtained are shown using dendrograms calculated with the Ward method and cluster analysis with Sneath’s dissimilarity criterion (Sneath & Sokal, 1973).

All the statistical analyses, which are described above, were performed with the use of STATISTICA 12.0 software (StatSoft, Inc., Tulsa, OK, USA).

## Results

### Fungal D2 LSU rRNA and ITS1 analysis

**Figure 1:**
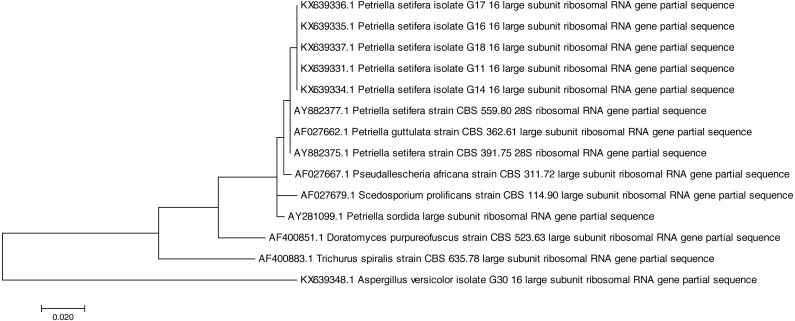
Phylogenetic tree based on the D2 region of LSU rRNA sequences of *Petriella setifera* strains.

**Figure 2:**
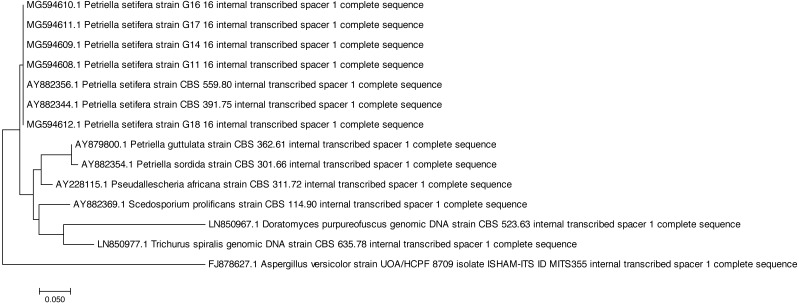
Phylogenetic tree based on the ITS1 sequences of *Petriella setifera* strains.

Both of the sequencing results confirmed that all of the tested strains were identified as *Petriella setifera* and that they were separated by another known species at the genus level (Fig. 1), as shown by the analysis of their D2 LSU rRNA; whereas the ITS1 analysis has separated the strains as a function of the species (Fig. 2).

In fact, the phylogenetic analysis showed a clear separation of all isolates into the clusters. For LSU analysis, we observed a separation in two clusters. The first group included the species *Aspergillus versicolor* strain G30, whereas the other comprised the species belonging to the Microascaceae family (i.e., *Petriella* sp., *Trichurus spiralis* strain CBS 635.78, *Doratomyces purpureofuscus* strain CBS 523.63, *Scedosporium prolificans* strain CBS 114.90, and *Pseudallescheria africana* strain CBS 311.72). Furthermore, in the latter subgroup described above, the sequencing of the D2 LSU region did not lead to a clear separation of the strains of *Petriella setifera* and *P. guttulata*.

On the other hand, for the ITS1 analysis (Fig. 2) we observe a separation into three clusters. The first group (as for the previous analysis) included the *Aspergillus versicolor*. In the second group, we found a species which belongs to the Microascaceae family (*Trichurus spiralis*, *Doratomyces purpureofuscus*, *Scedosporium prolificans*, *Pseudallescheria africana*, *Petriella sordida* and *P. guttulata*). Whereas in the final cluster, we found our isolated five strains plus the two published ITS1 sequences of *Petriella setifera*.

### AFLP fingerprinting analysis

**Figure 3:**
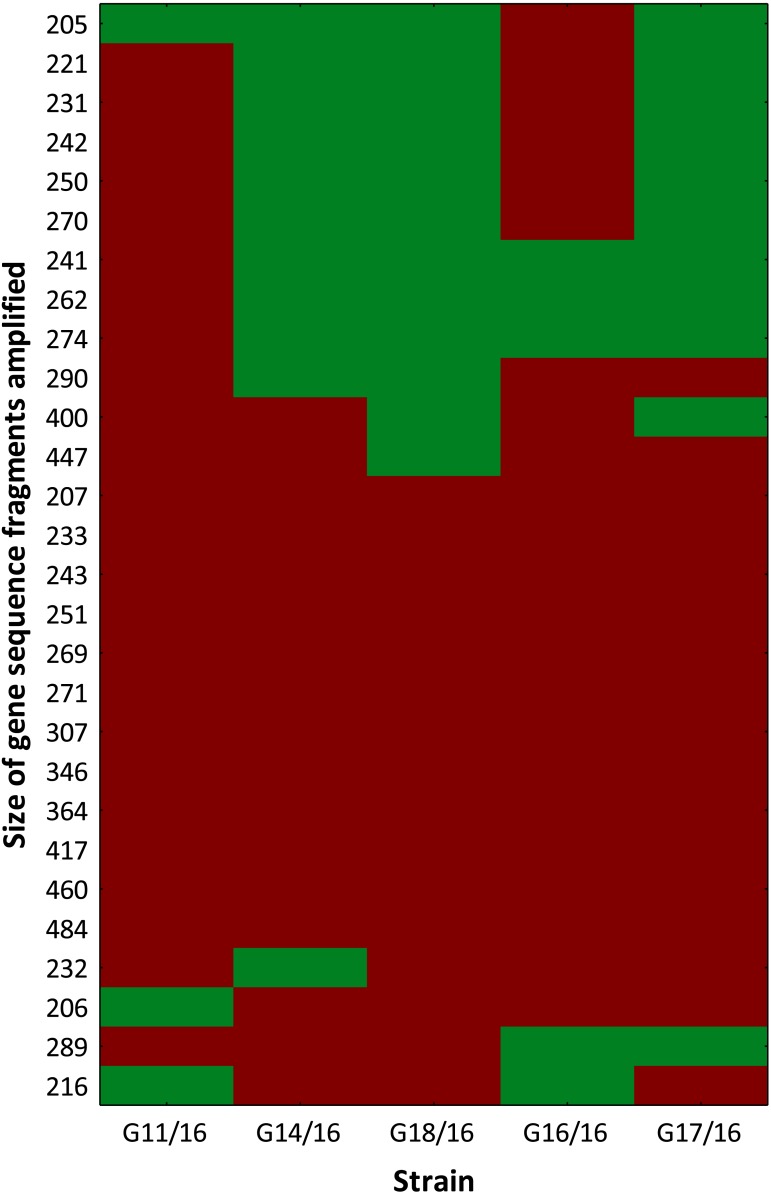
Genotype profile of *Petriella setifera* strains. The colour scale at the heatmap indicates the presence (red) or absence (green) of the polymorphic peaks in each analysed strain.

The selective primers used in this analysis produced representative electropherograms. In this way, fluorescent AFLP banding between *Petriella setifera* isolates were revealed (Fig. S1). The findings revealed the presence of 28 polymorphic peaks in total with a minimum size of 205 bp and a maximum size of 484 bp, including 4 monomorphic peaks (14.29%), and only 12 of a total of 46 peaks (42.86%) were similar to all of the analysed isolates (Fig. 3).

**Figure 4:**
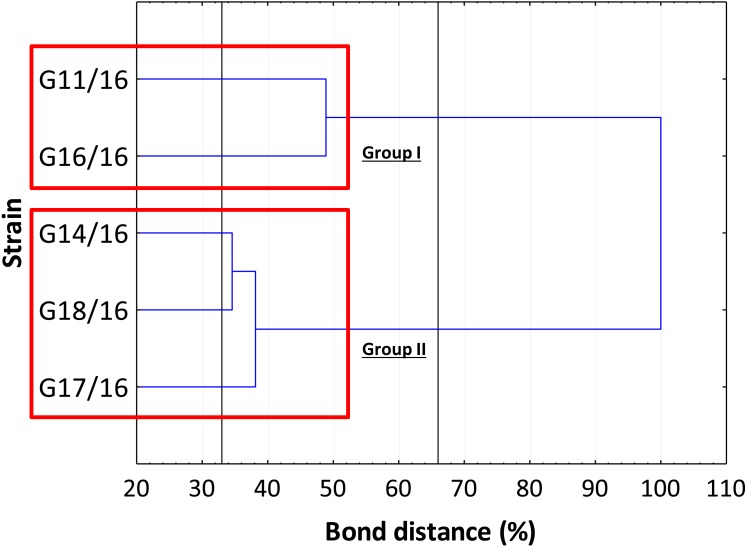
The dendrogram of *Petriella setifera* strains. This analysis depends on the presence or absence of the polymorphic peaks analysed through the AFLP analysis.

The genetic relationship between the isolates was demonstrated by the dendrogram (Fig. 4). The subdivision of all isolates is in accordance with the less restrictive Sneath criterion (66%). The isolates exhibited the following percentage of similarity: isolates G11/16 and G16/16 51% DNA profile similarity; isolates G17/16, G14/16, and G18/16 62% DNA profile similarity. In turn, at 33% of Sneath‘s restrictive criterion, we noted a separation between all the tested isolates. Moreover, through this analysis, we observed that four monomorphic peaks were present in just one strains.

### Functional diversity using the BIOLOG system

**Figure 5:**
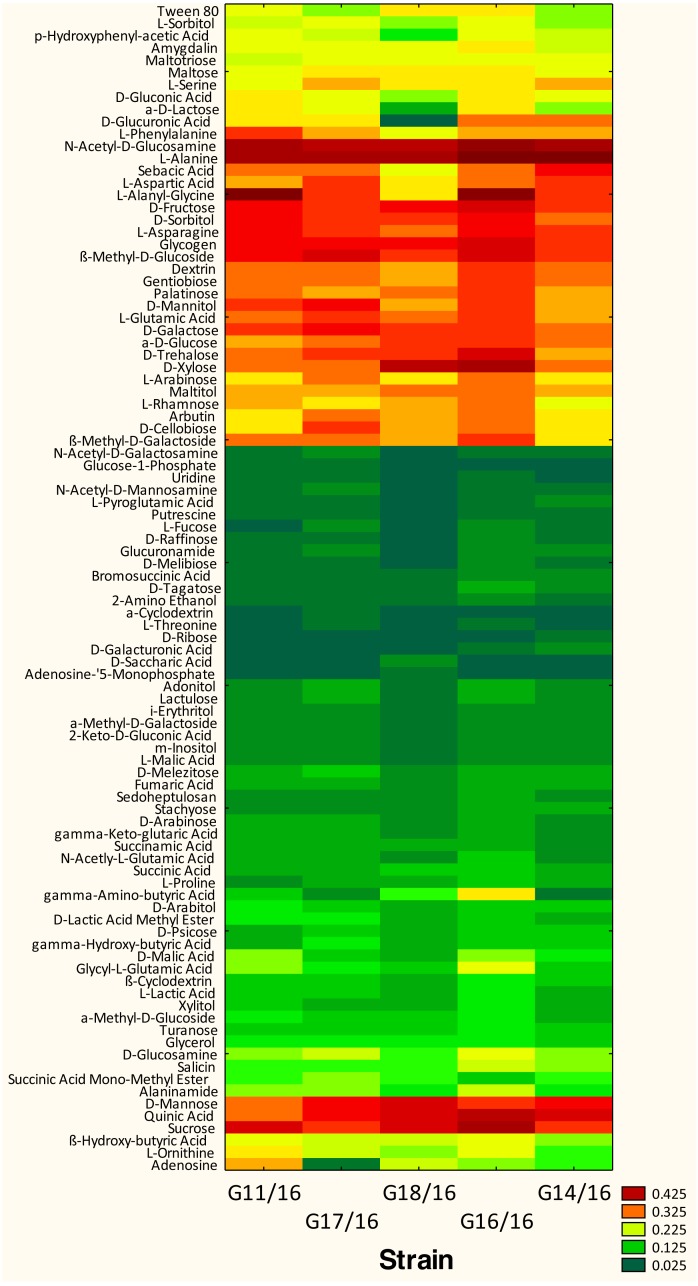
Phenotype profile of *Petriella setifera* strains. Colour scale of the heatmap indicates the growth of the organism (mycelial density measured at *A*_750nm_) in carbons substrate for each analysed strain during the experiment.

**Figure 6:**
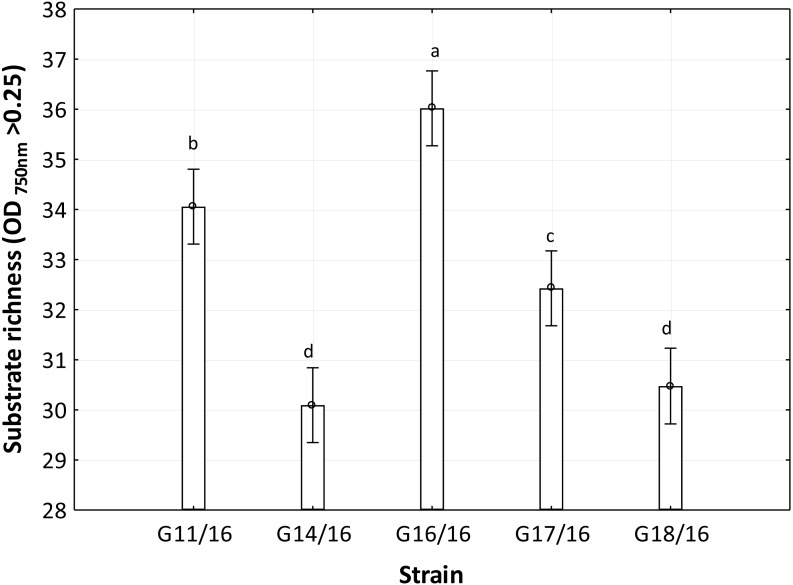
Functional diversity of *Petriella setifera* strains explained by the substrate richness (R) index. The vertical bars indicate the confidence intervals at 0.95 and the lowercase letters indicate the significant difference (*p* < 0.5) between each strain calculated through the post hoc Tukey test.

**Figure 7:**
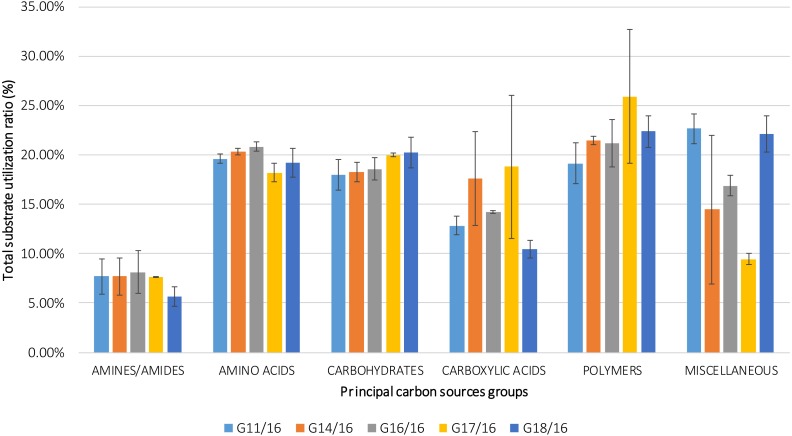
Percentage of total carbon source utilization for *Petriella setifera* strains. The carbon source utilization was drawn as a function of the principal six carbon sources groups (AMINES/AMIDES, AMINO ACIDS, CARBOXYDRATES, CARBOXYLIC ACIDS, POLYMERS and MISCELLANEOUS). The vertical bars represent the deviation standard.

The utilization profiles of carbon sources by these isolates revealed a broad intraspecific variability (Fig. 5). Significant differences (approximately up to 6 times) were demonstrated in the substrate richness (R) index and especially, we observed that the strains G16/16, G11/16 and G17/16 presented a significantly different substrate richness between them and between the two remaining strains (G14/16 and G18/16) (Fig. 6). These findings were supported by an ANOVA analysis and the post hoc Tukey test. Through the ANOVA analysis, we found that the strain, the incubation time and the interaction between these two factors had significant effect (*p* < 0.05) on the substrate richness (Table S1). All the five strains used an average of 92% of the 95 available carbon substrates; in particular, they used more carbohydrate sources (average of 95.45% of the total 44 analysed substrates). In total, each strain utilised more amino acid, carbohydrate and polymer; but for the total utilization of carboxylic acid and miscellaneous, we observed a different utilization patterns between the strains (Fig. 7).

We found that all of the *P. setifera* strains were extensively capable of metabolizing the carbon substrates at relatively high levels, especially carbohydrates (i.e., N-Acetyl-D-Glucosamine, D-Fructose, D-Galactose, D-Mannose, β-Methyl-D-Glucoside, D-Sorbitol, Sucrose and D-Xylose), one polymer (i.e., Glycogen), one carboxylic acid (i.e., Quinic Acid), and two amino acids (i.e., L-Alanine and L-Asparagine) (Fig. 5). Furthermore, we found that a few substrates were not used by the analysed strains. For example, *P. setifera* G18/16 did not metabolize N-Acetyl-D-Galactosamine, N-Acetyl-D-Mannosamine, α-Cyclodextrin, L-Fucose, D-Galacturonic Acid, Glucose-l-Phosphate, Glucuronamide, D-Glucuronic Acid, D-Melibiose, D-Raffinose, D-Ribose, L-Pyroglutamic Acid, L-Threonine, Putrescine and Uridine, but it metabolized two substrates (D-Saccharic Acid and Adenosine-5′-Monophosphate), which were not utilized by the following isolates; G11/16, G17/16, G16/16 and G14/16 (Fig. 5).

**Figure 8:**
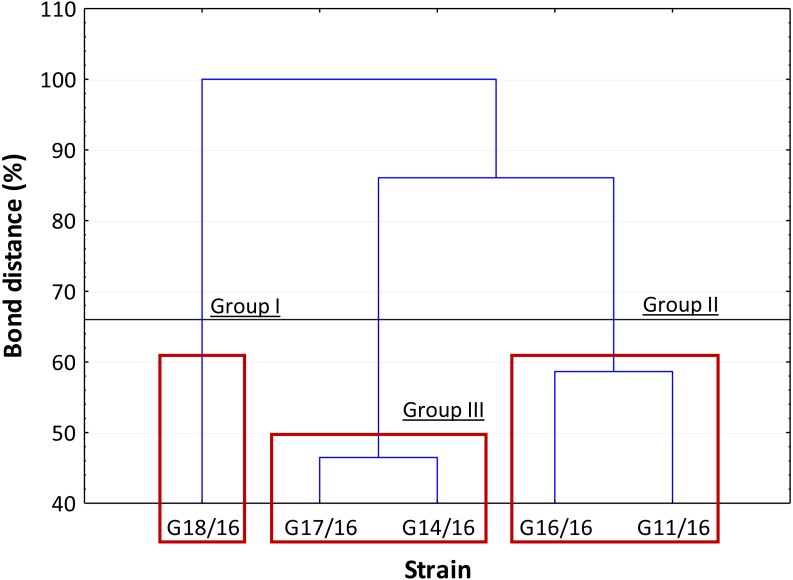
Cluster analysis between *Petriella setifera* strains. The cluster analysis depending on the carbon source utilization located inside BIOLOG FF Plates™.

The dendrogram showed that the strains were separated into three clusters, in accordance with Sneath’s dissimilarity criterion (66%) (Fig. 8). The first group included the isolate G18/16 with a metabolic profile similarity of 0%, the second one consisted of isolates G16/16 and G11/16, and the third one included the G17/16 and G14/16 isolates with a metabolic profile similarity of 42% and 54%, respectively.

The fungal activity, expressed by the AWDD (Average Well Density Development) index, was analysed through a two-way ANOVA and we found that the type of strain, incubation time and the interaction between these two factors significantly affected the AWDD index (Table S2 and Fig. S2). The same index was analysed through a two-way ANOVA as a function of the principal six carbon sources groups, but it was not affected by the interaction of strain and incubation time (Tables S3–S8).

The AWDD index has provided us with a further information concerning the analysed strains. In the Fig. 5 it was possible to observe how the five strains had approximately the same fungal activity until the 120-incubation time (h). After this time, we may observe an increase in the fungal activity only for the G16/16 until the end of the experiment.

## Discussion

To identify the isolated fungal species, we have used the Large Subunit Ribosomal (LSU rDNA) and Internal Transcribed Spacer (ITS) sequencing. According to many authors (Schoch et al., 2012; Pawlik et al., 2015a; Pawlik et al., 2015b), to identify a Fungus, it is possible to apply the ITS sequencing because it is a standard fingerprint for fungi (Brown, Rigdon-Huss & Jumpponen, 2014; Porras-Alfaro et al., 2014). The DNA sequences of D1-D2 hypervariable regions encoded from LSU rDNA and the ITS regions contain conservative and variable sequence regions. There have been studies on the identification of fungi by comparing the use of ITS and LSU regions. Brown, Rigdon-Huss & Jumpponen (2014), Porras-Alfaro et al. (2014) and Arbefeville, Harris & Ferrieri (2017) found that the accuracy and specificity of fungal identification techniques based on ITS and LSU rDNA regions produced similar results. Moreover, Liu et al. (2012) created a fungal database of the LSU gene and, progressively, this was used to track fungal composition in different locations and responses to environmental perturbations (Weber, Vilgalys & Kuske, 2013; Steven et al., 2014). Zhao, Wang & Wei (2013) claimed that the identification of fungi performed with a highly-conserved region was not phylogenetically informative within the family level. In fact, Issakainen et al. (1999) and Issakainen et al. (2003) developed a taxonomic classification using the LSU and the Small Subunit Ribosomal (SSU) rRNA gene, and they confirmed that both of these two regions may be used for phylogenetic analysis. In particular, the SSU rRNA is better to suited to analysing the higher taxonomic level, whereas the LSU rRNA is better for analysing closely related genera (Issakainen et al., 1999).

All of the analysed strains may be regarded as *Petriella setifera* (Fig. 1), as revealed in the phylogenetic tree and in particular it may be confirmed that the five fungal isolates were unknown until we published the partial genome for these strains (D2 LSU, ITS) in GenBank. The LSU rRNA sequencing provided an explanation for the good separation between the other genera belonging to the Microascaceae family, but this approach did not reveal any significant differences within the *Petriella* sp.. In fact, to reveal the separation of strains at the family level in the fungal domain, the sequencing of the LSU region should be carried out. Christ et al. (2011) revealed that to view the differences within a family, the best course of action is to sequence the ITS region because of its high variability and resolution at the species level. This was also confirmed by the phylogenetic study on the Microascaceae family performed by Lackner et al. (2014). In our study, the lack of intraspecific variability was found through the sequencing of LSU rRNA and ITS1.

To analyse the genetic diversity of *Petriella setifera*, the Amplified Fragment Length Polymorphisms (AFLP) analysis was used. The AFLP analysis was first described by Vos et al. (1995) and it was used to analyse a fungal community at the taxonomic level by Majer et al. (1996). This fingerprinting analysis consists of three principal steps: restriction of the total genomic DNA and ligation to oligonucleotide adapters, the selective amplification of restricted fragments, and the analysis of the amplified fragments through vertical electrophoresis in a polyacrylamide gel or using the capillary sequencing approach in a genetic analyser. The AFLP facilitates an estimation of the genetic diversity (Mueller & Wolfenbarger, 1999) and the levels of intraspecific variation (Tooley et al., 2000) between and within species owing to its taxonomic range, discriminatory power, reproducibility, lack of any need for knowledge concerning the nucleotide sequence, and ease of interpretation and standardization (Savelkoul et al., 1999; Perrone et al., 2006a). This was confirmed by the recent studies of Perrone et al. (2006a) and Perrone et al. (2006b), in which the AFLP was used to clarify the relationship within or between closely related species. The application of AFLP analysis to fungal studies has also been demonstrated by other authors (Bakkeren, Kronstad & Lévesque, 2000; Tooley et al., 2000; Abdel-Satar et al., 2003; Radišek et al., 2003; Schmidt, Niessen & Vogel, 2004; Perrone et al., 2006a; Perrone et al., 2006b; Pawlik et al., 2015a; Pawlik et al., 2015b; Rola et al., 2015).

The functional diversity, i.e., the fungal ability to use different carbon sources, is assessed by means of the BIOLOG FF MicroPlates™ method. This system is a rapid method for analysis of the catabolic potential of a fungal community or fungal strain pure culture based on their abilities to utilize 95 carbon substrates. Based on the results of catabolic profiles, we can determine two ecological indices (i.e., substrate richness (R) and Average Well Density Development (AWDD)) that may help us to understand and know the role of fungi. These indices are especially sensitive indicators that reveal the differences between the strains (Frąc, Oszust & Lipiec, 2012). In the last year, the BIOLOG system was introduced for the rapid characterization of the fungal community (Stefanowicz, 2006; Singh, 2009; Janusz et al., 2015; Pawlik et al., 2015a; Pawlik et al., 2015b; Rola et al., 2015). Recently, three studies of fungal species have been carried out using BIOLOG FF Plate™ and AFLP fingerprinting analysis; in the first one, Rola et al. (2015) used these two methodologies to analyse the phenotypic and genetic diversities of *Aspergillus* strains which synthesize glucose dehydrogenase. The other two studies estimated the genetic and metabolic biodiversities in *Ganoderma lucidum* strains (Pawlik et al., 2015a) and in *Coprinus comatus* (Pawlik et al., 2015b). The results of Xie et al. (2011) indicated that genetic microbial diversity are largely influenced by environmental factors which is very important for understanding of metabolic potential of microorganisms.

However, to the best of our knowledge, there are no reports describing the genetic and functional diversities of *Petriella setifera* through AFLP fingerprinting and BIOLOG FF Plates™. In the last 15 years many researchers have used the AFLP analysis to identify the intraspecific variability within a fungal species (Bakkeren, Kronstad & Lévesque, 2000; Tooley et al., 2000; Abdel-Satar et al., 2003; Radišek et al., 2003; Schmidt, Niessen & Vogel, 2004; Perrone et al., 2006a; Perrone et al., 2006b; Pawlik et al., 2015a; Pawlik et al., 2015b; Rola et al., 2015)), and 7 years ago they began to use BIOLOG methodology to estimate the functional diversity (Feng et al., 2009; Singh, 2009; Albrecht et al., 2010; Shengnan et al., 2011; Lucas et al., 2013; Janusz et al., 2015; Pawlik et al., 2015a; Pawlik et al., 2015b; Rola et al., 2015; Panek, Frąc & Bilińska-Wielgus, 2016). All of these investigations explain the validity and suitability of using these methodologies to discover the intraspecific differences between fungal species at a genetic and functional level.

An analysis of the metabolic potential has revealed the presence of intraspecific variability within the *P. setifera* strains and differences were found in the affinity and modality in the use of these carbon substrates. When we analysed the dendrogram of the patterns of carbon sources utilization (Fig. 8), we noted that the subdivision into the three clusters was a function of the utilization of these substrates. Strains G16/16, G11/16 and G17/16 metabolized more substrates than the others, and this was confirmed by the high substrate richness index (R index, Fig. 6). Another aspect that distinguishes the *P. setifera* strains in the functional diversity was the different pattern of substrate utilization between the isolates. Figure 7 showed clearly that cluster G11/16 and G16/16 used the five principal carbon source groups in the same way, which was completely different from cluster G14/16 and G17/16; in fact, these clusters exhibited a metabolic profile similarity of 42% and 54%, respectively (Fig. 8). We observed that the strain G18/16 utilized these carbon substances in a different way from the other two groups, in particular we observed a different utilization pattern for carboxylic acid and miscellaneous (Fig. 7). Moreover, Fig. 7 demonstrated that all of the strains were characterized by a different C-substrate utilization ratio, especially for carboxylic acids, polymers, and miscellaneous substrates, whereas the patterns for the other three groups (i.e., amines/amides, amino acids, and carbohydrates) were the same for all of the strains. The results of the BIOLOG FF Plates™ analysis indicated intraspecific differences in the phenotypic profiles. This means that these isolates have different metabolic abilities to degrade the analysed carbon sources. These findings were confirmed by an analysis of the density of each isolate. The AWDD showed that this measure for all of the analysed isolates increased after the 24 h of incubation and it remained higher throughout the time of incubation. At the beginning of the experiment (before the 24 h of incubation) all the five strains had the same lower fungal activity and after this point, we observe a larger increase (an exponential phase) of the activity for all strains from 48 to 72 incubation hours. From 72 to 120 incubation hours, the analysed strains showed equal activity (similar a plateau situation). After this moment, only for 144 and 192 h of the incubation, we observed that four of the analysed strains had the same activity and only the G16/16 strain presented an increment in activity until the end of the experiment. These modifications of the fungal activity mean that at moment when the strains come into contact with the carbon substrates, they display a lower fungal activity level followed by an exponential phase. The final phase may be the phase in which the substrates are degraded most. In the last 120 incubation hours, we observe a plateau phase due to the possible limitation of the substrate amount or the excessive presence of the inhibitor products. The significantly different behaviours in the fungal activity between the five strains, were observed at 144 and 192 incubation hours (Fig. S2).

The *Petriella setifera*, which may be found in decaying wood, belongs to soft rot fungi that degrade cellulose and hemicellulose. We found that all of the isolates degraded the substances that can be produced during hemicellulose degradation at high level (i.e., D-Arabinose, L-Arabinose, D-Glucuronic Acid, Xylitol, γ-Amino-Butyric Acid, D-Mannose, D-Xylose and L-Rhamnose) or during cellulose degradation (i.e., α-D-Glucose and D-Cellobiose). These results were associated with the properties of soft-rot fungi (Martínez et al., 2005; Schwarze, 2007; Mathieu et al., 2013). Furthermore, we noted that all the analysed isolates degraded Quinic Acid at a high level, this substance is involved in the synthesis of the S- and G-type of lignin (Albrecht et al., 2010; Hatakka & Hammel, 2011). This may suggest the possible involvement of *P. setifera* in the partial degradation of lignin, which is in accordance with the findings reported by other researchers (Hammel, 1996; Schwarze, 2007; Janusz et al., 2013; Mathieu et al., 2013). In conclusion, the results of the BIOLOG FF Plates™ analysis have demonstrated a great intraspecific variability of the analysed *P. setifera* strains.

The findings obtained with the use of AFLP fingerprinting analysis confirmed the presence of genetic variability within the isolates of *Petriella setifera*. It is evident in Fig. 4 that the dendrogram based on cluster analysis divides the analysed strains into two groups (in accordance with Sneath’s dissimilarity criteria of 66%). However, at a 33% dissimilarity coefficient, the analysed strains are not related to each other. This differentiation was made as a function of the number of detected polymorphisms. The cluster with G11/16 and G16/16 had a 52% of AFLP profile similarity, since these two isolates exhibited in total an average of 24 common peaks from a total of 27 polymorphic peaks and five polymorphic peaks were not observed in the other strains. The cluster with G14/16, G18/16, and G17/16 had a 62% AFLP profile similarity with an average of 17 common peaks from a total 19 polymorphic peaks, there was only one common peak, which was not detected in the previous cluster. This means that more polymorphism peaks were detected in the cluster with G11/16 and G16/16 than in the other strains. The results of AFLP analysis confirm that this new protocol has successfully differentiated between the isolated *P. setifera* strains.

In general, the results of grouping obtained from AFLP and BIOLOG FF Plates™ analyses revealed differences in the graphs (Figs. 4 and 8). The BIOLOG and AFLP are proper tools, to evaluate intraspecific variability among isolates as proved in our experiments, which is also consistent with the findings of other authors (Bakkeren, Kronstad & Lévesque, 2000; Tooley et al., 2000; Abdel-Satar et al., 2003; Radišek et al., 2003; Schmidt, Niessen & Vogel, 2004; Stefanowicz, 2006; Perrone et al., 2006a; Perrone et al., 2006b; Singh, 2009; Janusz et al., 2015; Pawlik et al., 2015a; Pawlik et al., 2015b; Rola et al., 2015). When we analysed the two dendrograms obtained in the BIOLOG and AFLP analyses, we found that the clustering was completely different except for the cluster of isolates G16/16 and G11/16. Therefore, isolates G16/16 and G11/16 show more variability in the genetic and metabolic patterns because of the lower similarities in the DNA and metabolic profiles. Isolate G18/16 presented a metabolic profile similarity of 0% (Fig. 8) and this resulted in an initial separation of this strain from the other four isolates, due to the lower utilization of carbon substrates (80/95, 84.21%) and the lower substrate richness values (R index; Fig. 6). For the DNA profile (Fig. 4), strain G18/16 had a profile similarity of 66% (it was clustered with strain G14/16), as suggested by the detection of only 16 polymorphism peaks for this strain (16/28, 57.14%). For this reason, strain G18/16 displays a lower variability in the genetic and metabolic profiles. Finally, isolate G17/16 had a metabolic profile similarity of 54%, which was similar to strain G14/16, given their similar pattern of carbon substances utilization (Fig. 8). Regarding the DNA profile, G17/16 exhibited a similarity of 62%, which separated it from the cluster of isolates G14/16 and G18/16. We found that this separation between G17/16 and the latter cluster was revealed by the number of polymorphic peaks in common (14 out of a total of 19); additionally, a peak that was not present in the others two strains (G14/16 and G18/16) was detected for isolate G17/16. The differences obtained from the clustering of the AFLP and BIOLOG results, suggest that from a genetic and functional point of view the analysed strains show an intraspecific diversity at a level of the polymorphisms and the metabolic potential. Furthermore, these results may explain how these analysed strains can respond differently to the exposure to different carbon resources and from the genetic point of view. What is more, such results may indicate how the presence of polymorphisms could be related to the production of secondary metabolites. The results address the possibility that genetic diversity may not precisely overlap with the functional diversity. This may be an indication of the fungal dynamics and their responses to environmental conditions and the ecology of coprophilous organisms.

## Conclusions

This is the first report concerning the genetic and metabolic diversity of *Petriella setifera* strains isolated from industrial compost and the first description of a protocol for the AFLP fingerprinting analysis optimized for these fungal species. Using these two methodologies we have found the existence of intraspecific variability within the *Petriella setifera* strains at functional and genetic levels and these findings confirm that the two methodologies described in this study allow us to identify and elucidate the intraspecific diversity in DNA and metabolic profiles of previously unknown species. The results indicated that *P. setifera* strains are able to degrade substrates produced in the degradation of hemicellulose (D-Arabinose, L-Arabinose, D-Glucuronic Acid, Xylitol, γ-Amino-Butyric Acid, D-Mannose, D-Xylose and L-Rhamnose), cellulose (α-D-Glucose and D-Cellobiose) and the synthesis of lignin (Quinic Acid) at a high level, showing their importance in ecosystem processes as decomposers of carbon compounds and as organisms, which make a significant contribution to carbon cycling in the ecosystem. Nevertheless, further studies are required, especially those focused on the genetic and metabolic aspect of this species, since there are insufficient data on the utilization of the carbon sources from different organic wastes containing e.g., cellulose, hemicellulose, and lignin. This analysis could shed light on the degradation pathway of cellulose and hemicellulose by *P. setifera*. The results may help us to recognize whether these species are able to degrade lignin similar to soft rot-fungi, which carry out a partial degradation of this substance, and to clarify whether this fungus may be included in the group of brown rot fungi or only in the soft-rot fungi. Genetic analyses show that *Petriella setifera* clusters into two groups, but metabolic analyses indicate three groups. Based on these results, genetic diversity does not always correlate with the level of functional diversity. It is important to understand ecosystem functioning and the usefulness of strains for environmental applications, especially for their metabolic abilities rather than genetic diversities.

##  Supplemental Information

10.7717/peerj.4420/supp-1 Supplemental Information 1 AFLP results

10.7717/peerj.4420/supp-2 Figure S1 DNA fingerprinting *Petriella setifera* strains based on Amplified Fragment Length Polymorphism

10.7717/peerj.4420/supp-3 Figure S2 The growth of the analysed strains on the different carbon substrates during 192 hours of incubation The growth of these fungal strains was explained by Average Well Density Development (AWDD) index. The vertical bars indicate the confidence intervals at 0.95. Each incubation hour was analysed by a two-way ANOVA and the post hoc Tukey test. The lower-case letters above each column describe the statistical difference between the treatments (*p* < 0.05).

10.7717/peerj.4420/supp-4 Supplemental Information 2 FF results

10.7717/peerj.4420/supp-5 Table S1 *Petriella setifera* strains responses to substrates richness index (R) The incubation time and strain effects on the substrate richness index (R) were determined by two-way ANOVA.

10.7717/peerj.4420/supp-6 Table S2 *Petriella setifera* strains responses to Average Well Density Development index (AWDD) The incubation time and strain effects on the Average Well Density Development index (AWDD) were determined by two-way ANOVA.

10.7717/peerj.4420/supp-7 Table S3 *Petriella setifera* strains responses to amine/amides (Biolog FF Plate analysis) The incubation time and strain effects on the Average Well Density Development index (AWDD) of the strains incubated with amine/amides sources.

10.7717/peerj.4420/supp-8 Table S4 *Petriella setifera* strains responses to amino acids (Biolog FF Plate analysis) The incubation time and strain effects on the Average Well Density Development index (AWDD) of the strains incubated with amino acids sources.

10.7717/peerj.4420/supp-9 Table S5 *Petriella setifera* strains responses to carbohydrates (Biolog FF Plate analysis) The incubation time and strain effects on the Average Well Density Development index (AWDD) of the strains incubated with carbohydrates sources.

10.7717/peerj.4420/supp-10 Table S6 *Petriella setifera* strains responses to carboxylic acids (Biolog FF Plate analysis) The incubation time and strain effects on the Average Well Density Development index (AWDD) of the strains incubated with carboxylic acids sources.

10.7717/peerj.4420/supp-11 Table S7 *Petriella setifera* strains responses to polymers (Biolog FF Plate analysis) The incubation time and strain effects on the Average Well Density Development index (AWDD) of the strains incubated with polymers sources.

10.7717/peerj.4420/supp-12 Table S8 *Petriella setifera* strains responses to miscellaneous (Biolog FF Plate analysis) The incubation time and strain effects on the Average Well Density Development index (AWDD) of the strains incubated with miscellaneous sources.

## Additional Information and Declarations

### DNA Deposition

The following information was supplied regarding the deposition of DNA sequences: The sequences described here are accessible via GenBank accession numbers:
KX639331, KX639334, KX639335, KX639336, KX639337, MG594608.1, MG594609.1, MG594610.1, MG594611.1 and MG594612.1.

### Data Availability

The following information was supplied regarding data availability: The raw data is provided in the Supplemental Files.
