# Supplementary figures and images for: Intraspecific functional and genetic diversity of Petriella setifera
Source: PeerJ. 2018 Feb 28;6:e4420. doi: 10.7717/peerj.4420 (PMC5834937; doi:10.7717/peerj.4420)

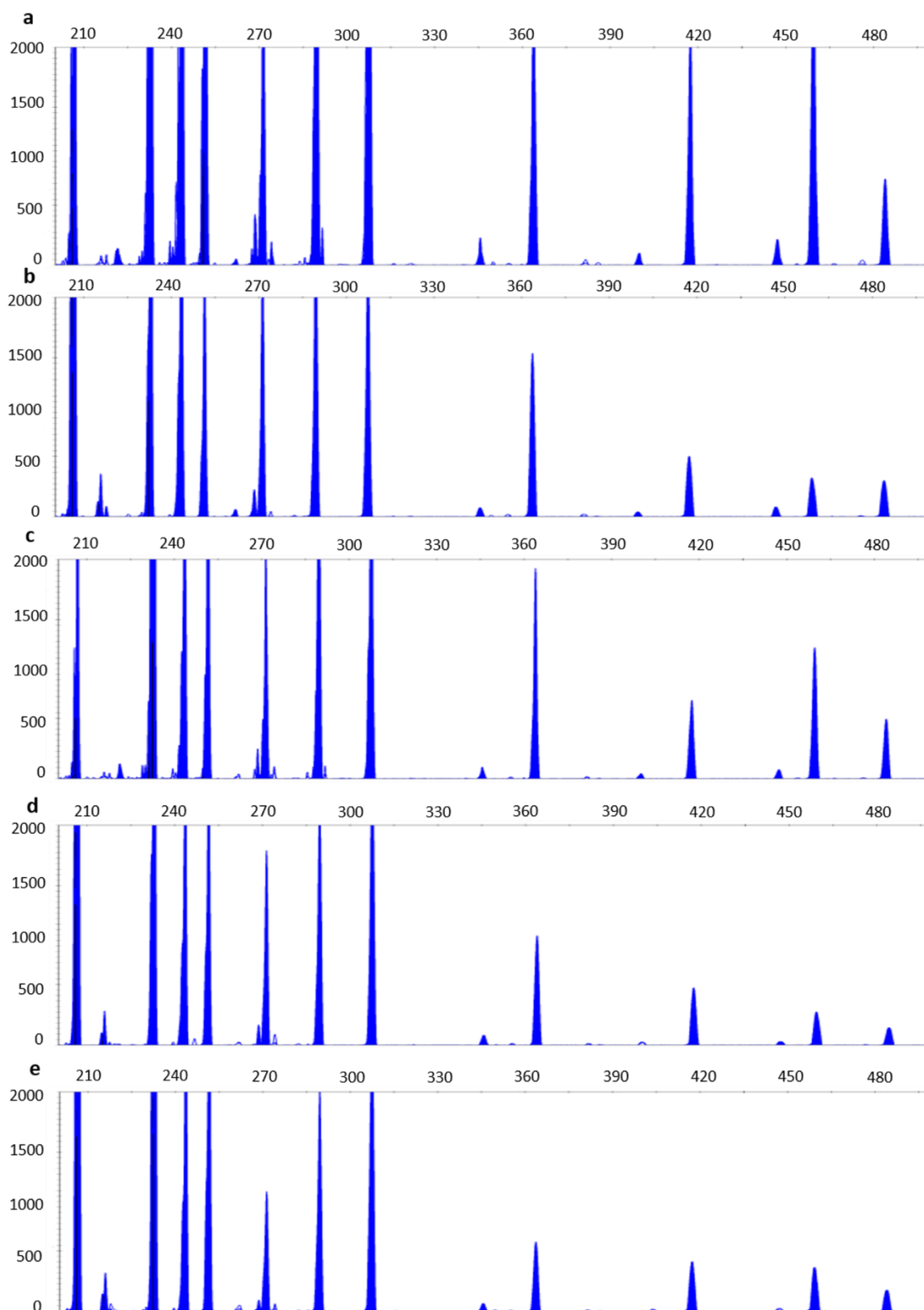

Supplement: Figure S1 [file peerj-06-4420-s002.pdf]

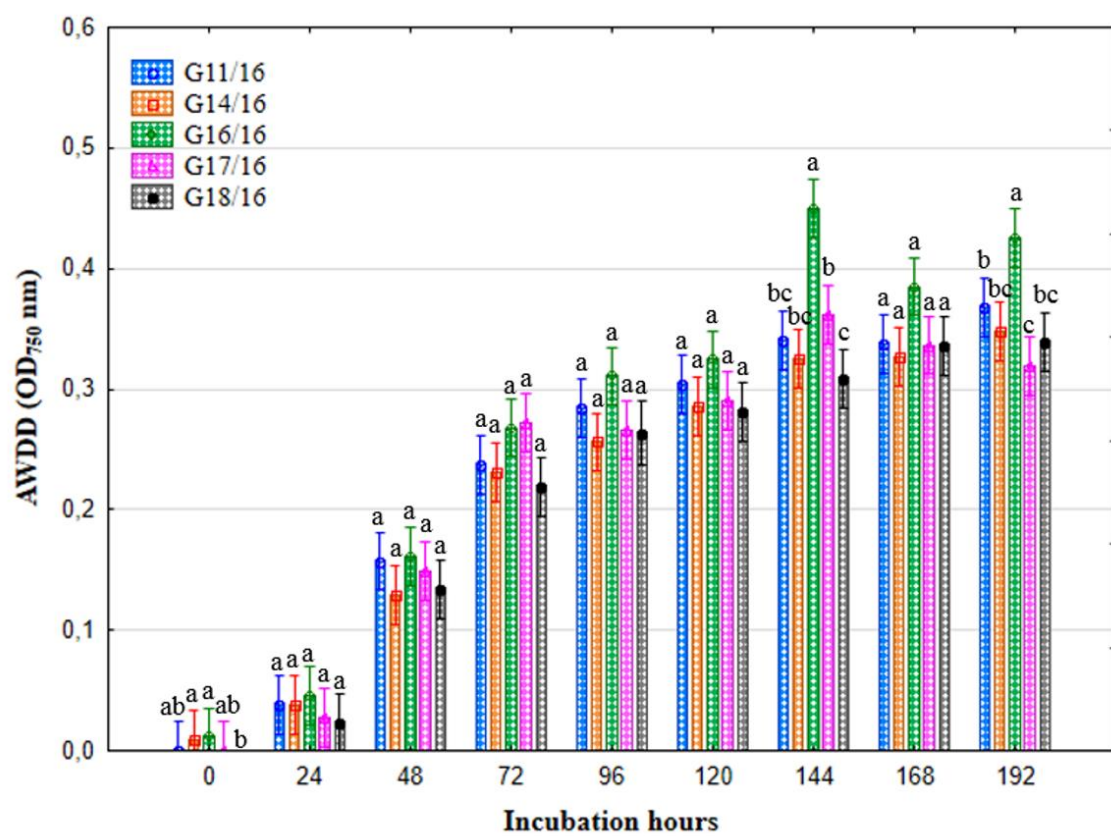

Supplement: Figure S2 — The growth of these fungal strains was explained by Average Well Density Development (AWDD) index. The vertical bars indicate the confidence intervals at 0.95. Each incubation hour was analysed by a two-way ANOVA and the post hoc Tukey test. The lower-case letters above each column describe the statistical difference between the treatments (p < 0.05). [file peerj-06-4420-s003.pdf]
