# Supplementary material for: Intraspecific functional and genetic diversity of Petriella setifera
Source: PeerJ. 2018 Feb 28;6:e4420. doi: 10.7717/peerj.4420 (PMC5834937; doi:10.7717/peerj.4420)
Supplement: Table S1 — The incubation time and strain effects on the substrate richness index (R) were determined by two-way ANOVA. [file peerj-06-4420-s005.docx]

| Effect | df | Mean square | F | p |
| --- | --- | --- | --- | --- |
| Incubation time (h) | 8 | 11610.3 | 1495.92 | 0.000000 |
| Strain | 4 | 331.3 | 42.69 | 0.000000 |
| Incubation time * strain | 32 | 35.4 | 4.56 | 0.000000 |
| Residual | 224 | 7.8 |  |  |
