# Supplementary material for: Intraspecific functional and genetic diversity of Petriella setifera
Source: PeerJ. 2018 Feb 28;6:e4420. doi: 10.7717/peerj.4420 (PMC5834937; doi:10.7717/peerj.4420)
Supplement: Table S2 — The incubation time and strain effects on the Average Well Density Development index (AWDD) were determined by two-way ANOVA. [file peerj-06-4420-s006.docx]

| Effect | df | Mean square | F | p |
| --- | --- | --- | --- | --- |
| Incubation time (h) | 8 | 0.56126 | 623.74 | 0.000000 |
| Strain | 4 | 0.02395 | 26.62 | 0.000000 |
| Incubation time * strain | 32 | 0.00221 | 2.46 | 0.000070 |
| Residual | 224 | 0.00090 |  |  |
