# Supplementary material for: Intraspecific functional and genetic diversity of Petriella setifera
Source: PeerJ. 2018 Feb 28;6:e4420. doi: 10.7717/peerj.4420 (PMC5834937; doi:10.7717/peerj.4420)
Supplement: Table S4 — The incubation time and strain effects on the Average Well Density Development index (AWDD) of the strains incubated with amino acids sources. [file peerj-06-4420-s008.docx]

| Effect | df | Mean square | F | p |
| --- | --- | --- | --- | --- |
| Strain | 4 | 0.025346 | 14.758 | 0.000000 |
| Incubation time (h) | 8 | 0.406864 | 236.905 | 0.000000 |
| Incubation time * strain | 32 | 0.002366 | 1.378 | 0.158962 |
| Residual | 45 | 0.001717 |  |  |
