# Supplementary material for: Intraspecific functional and genetic diversity of Petriella setifera
Source: PeerJ. 2018 Feb 28;6:e4420. doi: 10.7717/peerj.4420 (PMC5834937; doi:10.7717/peerj.4420)
Supplement: Table S7 — The incubation time and strain effects on the Average Well Density Development index (AWDD) of the strains incubated with polymers sources. [file peerj-06-4420-s011.docx]

| Effect | df | Mean square | F | p |
| --- | --- | --- | --- | --- |
| Strain | 4 | 0.004799 | 3.682 | 0.000000 |
| Incubation time (h) | 8 | 0.114546 | 37.884 | 0.011203 |
| Incubation time * strain | 32 | 0.000581 | 0.446 | 0.990637 |
| Residual | 45 | 0.001303 |  |  |
