# Supplementary material for: Intraspecific functional and genetic diversity of Petriella setifera
Source: PeerJ. 2018 Feb 28;6:e4420. doi: 10.7717/peerj.4420 (PMC5834937; doi:10.7717/peerj.4420)
Supplement: Table S8 — The incubation time and strain effects on the Average Well Density Development index (AWDD) of the strains incubated with miscellaneous sources. [file peerj-06-4420-s012.docx]

| Effect | df | Mean square | F | p |
| --- | --- | --- | --- | --- |
| Strain | 4 | 0.003280 | 2.555 | 0.051655 |
| Incubation time (h) | 8 | 0.040247 | 31.350 | 0.000000 |
| Incubation time * strain | 32 | 0.000877 | 0.683 | 0.8694000 |
| Residual | 45 | 0.001284 |  |  |
